# Dysbiosis of gut microbiota in Polish patients with ulcerative colitis: a pilot study

**DOI:** 10.1038/s41598-021-81628-3

**Published:** 2021-01-25

**Authors:** Oliwia Zakerska-Banaszak, Hanna Tomczak, Marcin Gabryel, Alina Baturo, Lukasz Wolko, Michal Michalak, Natalia Malinska, Dorota Mankowska-Wierzbicka, Piotr Eder, Agnieszka Dobrowolska, Ryszard Slomski, Marzena Skrzypczak-Zielinska

**Affiliations:** 1 Institute of Human Genetics, Polish Academy of Sciences, Strzeszynska 32, 60-479 Poznan, Poland; 2 Central Microbiology Laboratory, H. Swiecicki Clinical Hospital, Poznan University of Medical Sciences, Poznan, Poland; 3 Department of Dermatology and Venereology, Poznan University of Medical Sciences, Poznan, Poland; 4 Department of Gastroenterology, Dietetics and Internal Diseases, Poznan University of Medical Sciences, Poznan, Poland; 5 Department of Biochemistry and Biotechnology, University of Life Sciences, Poznan, Poland; 6 Department of Computer Science and Statistics, Poznan University of Medical Sciences, Poznan, Poland

**Keywords:** Genetics, Microbiology, Molecular biology, Gastroenterology, Medical research

## Abstract

Ulcerative colitis (UC) is a chronic immune-mediated disorder, whose etiology is not fully understood and for which no effective treatment is available. Recently, research has focused on the dysbiosis of gut microbiome in UC. However, the results so far remain inconsistent and insufficient to understand the microbial component in UC pathogenesis. In this study, we determine specific changes in the gut microbial profile in Polish UC patients compared to healthy subjects for the first time. Using 16S rRNA gene-based analysis we have described the intestinal microbial community in a group of 20 individuals (10 UC patients and 10 controls). Our results after multiple hypothesis testing correction demonstrated substantially lower gut microbiome diversity in UC cases compared to the controls and considerable differences at the phylum level, as well as among 13 bacterial families and 20 bacterial genera (*p* < 0.05). UC samples were more abundant in *Proteobacteria* (8.42%), *Actinobacteria* (6.89%) and *Candidate Division TM7* (2.88%) than those of healthy volunteers (2.57%, 2.29% and 0.012%, respectively). On the other hand, *Bacteroidetes* and *Verrucomicrobia* were presented at a lower level in UC relative to the controls (14% and 0% vs 27.97% and 4.47%, respectively). In conclusion, our results show a reduced gut microbial diversity in Polish UC patients, a reduction of taxa with an anti-inflammatory impact and an increased abundance of potentially pathogenic bacteria.

## Introduction

Ulcerative colitis (UC) belonging to inflammatory bowel diseases (IBD) is characterized by chronic inflammation of the colorectal mucosa and systematic biochemical abnormalities^1,2^. Over the past few years, the morbidity of UC has constantly increased, affecting millions of people worldwide. Therefore, it has become a global public health challenge^3,4^. Unfortunately, the available treatment involving immunosuppressants, corticosteroids, aminosalicylates, biological agents, diet or surgery only relieves the symptoms^5–8^. The lack of effective treatment is certainly a consequence of the as yet not fully understood etiology of the disease. However, it is known that UC has a multifactorial background based on a defective intestinal barrier and an impaired immune response against luminal antigens, which is modified by environmental factors in genetically predisposed people^9–12^.

Up to now, more than 100 UC-associated genetic *loci* have been indicated. Studies have shown that these candidate *loci* are mostly involved in regulating the immune response^13^. On the other hand, current research reports changes in gut microbiota balance in UC patients, underlying the possible role of microbial flora in UC pathogenesis. Although it still remains unclear whether dysbiosis is a cause or consequence of intestinal inflammation, much research has shown that gut microbiota constitute a crucial element of the host immune system, gut-associated lymphoid tissue (GALT), and disturbances to it may affect various diseases^14,15^. Trillions of microbes forming a complex ecosystem in the human gut influence the functioning of host organism due to microbial metabolites, such as short-chain fatty acids (SCFAs), carbohydrates, vitamins and gases^16^. SCFAs (mainly butyrate, propionate and acetate) activate the G protein-coupled receptors, which are located on macrophages, dendritic cells and mast cells. They regulate, among other things, the expression of multiple genes, such as anti- and pro-inflammatory cytokines, hormones release, claudin-5 and occluding production influencing the integrity of the blood–brain barrier^17–19^. There is evidence that specific bacterial species (from *Clostridia* and *Bacteroides* genera) of gut microbiota play a role in mucosal immune homeostasis and inflammation, particularly in the regulation of T helper 1, 2, 17 (Th1, Th2, Th17) and T-reg cells release, which are responsible for the induction and inhibition of colonic inflammation^20^.

In healthy conditions, human gut microbiota is composed of around 400–500 bacterial species, of which over 90% belong to four major phyla, i.e. *Firmicutes*, *Bacteroidetes*, *Proteobacteria* and *Actinobacteria*^21–23^. Indeed, the implementation of the Human Microbiome Project allowed first standards to be set for each bacterial phyla abundance in healthy subjects^21^. However, later studies on healthy populations have verified this data and showed that *Firmicutes* dominate among bacterial phylas, and together with *Bacteroidetes* constitute almost 90% of intestinal microbiota^24^. Studies also revealed, that abundance values of each phyla are characterized by large variability, within the population, as well as between them^22,23^. In the Polish population gut microbial composition has been characterized only in men with obesity and cardiometabolic disorders, so far^25,26^. There is no data available for healthy individuals.

Research have proved that factors, such as the way of sample preparation, analysed region (V1-V9) of 16S rRNA gene or applied sequencing technology, may affect abundance of each microbial taxa^27,28^. Studies revealed that over 50 genera displayed a positive correlation with a given primer pair-mediated amplification efficiency, thereby indicating that the analysed region of 16S rRNA gene plays an important role in the generation of a bias in the determination of gut microbiota composition^27^.

In UC patients, changes concerning the diversity and composition of gut microbiota on different taxonomic levels compared to healthy subjects have been observed. The dysbiosis associated with UC often relies on a decrease in beneficial, commensal bacteria, particularly those belonging to *Firmicutes* and *Bacteroidetes* phyla, and an increase in pathogenic species from the *Enterobacteriaceae* family^29–32^. However, there is great variability of results across studies, with contradictory findings, hence an in-depth analysis of gut microbiota in UC patients is still needed^28^. A fully explained pathogenesis of UC could be a milestone in the development of effective targeted therapy, as faecal microbiota transplantation (FMT) has recently promised to be^33^. Our recent pilot investigation concerning FMT in UC patients has proved a strong correlation between specific gut bacteria and clinical parameters in the FMT procedure determining its effectiveness^34^. Thus detailed knowledge about the microbiome of a healthy population is needed and our analysis of UC microbiota in Polish patients will constitute an important basis for therapeutic options and further research on UC.

The purpose of the current work was to investigate the gut microbial composition for the first time in Polish UC patients relative to healthy subjects. In particular, we wanted to discover specific quantitative and qualitative changes occurring on different taxonomic levels in the gut microbiota compared to healthy volunteers. Description of intestinal microbial diversity in patients with UC aims at a better understanding of the disease's mechanism and it is necessary in order to improve FMT efficacy as an encouraging treatment approach. Our results we have verified performing a meta-analysis with the similar publicly available data.

## Results

### Characteristics of the studied groups

In the present study, we investigated gut microbial composition in a group of 10 Polish UC cases and 10 healthy subjects. All patients had active UC disease, wherein 4 (40%) presented a moderate and 6 (60%) a severe form of the disease (Table 1).

**Table 1:** Baseline characteristics of UC patients and control group.

Variable	Patients	Control group	*p*-value
Sex (female/male), n (%)	5/5 (50/50)	6/4 (60/40)	0.65
Age (years), mean ± SD	47.4 ± 17.4	45.2 ± 9.3	0.31
BMI, mean ± SD	22 ± 2.7	23.8 ± 3.6	0.42
Disease duration (years), mean, range	5.9 (3–10)	–	–
Range of disease, n (%)		–	–
Left-sided	4 (40)	–	–
Extensive colitis	4 (40)	–	–
Pancolitis	2 (20)	–	–
Disease severity, n (%)		–	–
Mild	0 (0)	–	–
Moderate	4 (40)	–	–
Severe	6 (60)	–	–
Treatment, n (%)		–	–
Immunosuppressants	4 (40)	–	–
Corticosteroids	4 (40)	–	–
Aminosalicylates	10 (100)	–	–

*p*-value calculated using *t*-test.

### Differences in microbiota composition among UC patients compared to healthy controls

**Figure 1:**
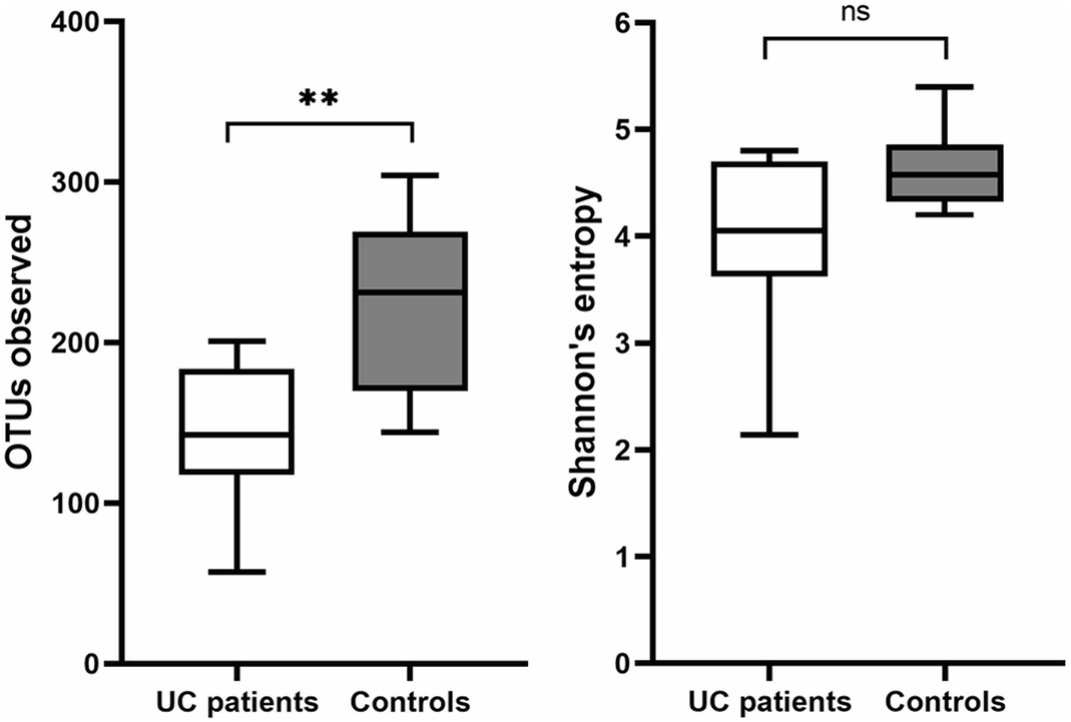
Microbial alpha diversity as OTU-richness and Shannon’s entropy in UC patients and healthy controls. *p*-value calculated using the non-parametric Mann–Whitney test, ^**^p < 0.01, ns—no significance.

First, we compared microbiota diversity in both groups of samples. The gut microbiome in our UC cases demonstrated significantly lower OTU-richness than in healthy individuals (*p* = 0.0029, Fig. 1). The mean OTU-richness observed for patients was 144, while for the controls it reached 225. However, alpha diversity, defined as Shannon’s entropy, did not reveal any significant differences between the patient and control groups, despite similar tendencies (*p* = 0.0622, Fig. 1).

**Figure 2:**
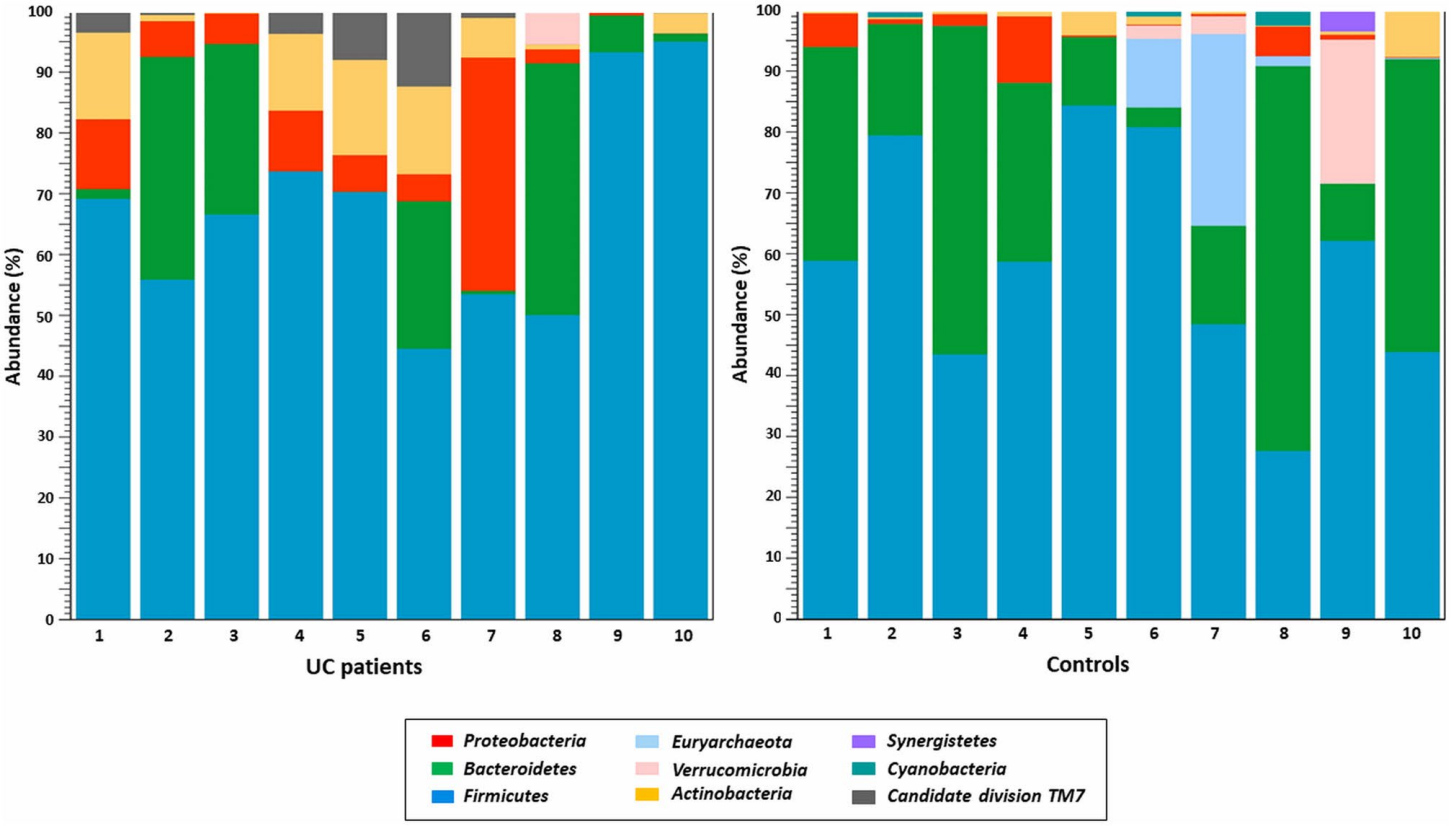
Individual profiles of gut microbial composition at the phylum level in the UC patient and control groups.

Profiles of gut microbial composition at the phylum level of the samples analysed reveal large inter-individual differences within the patient and control groups, as illustrated in Fig. 2.

**Figure 3:**
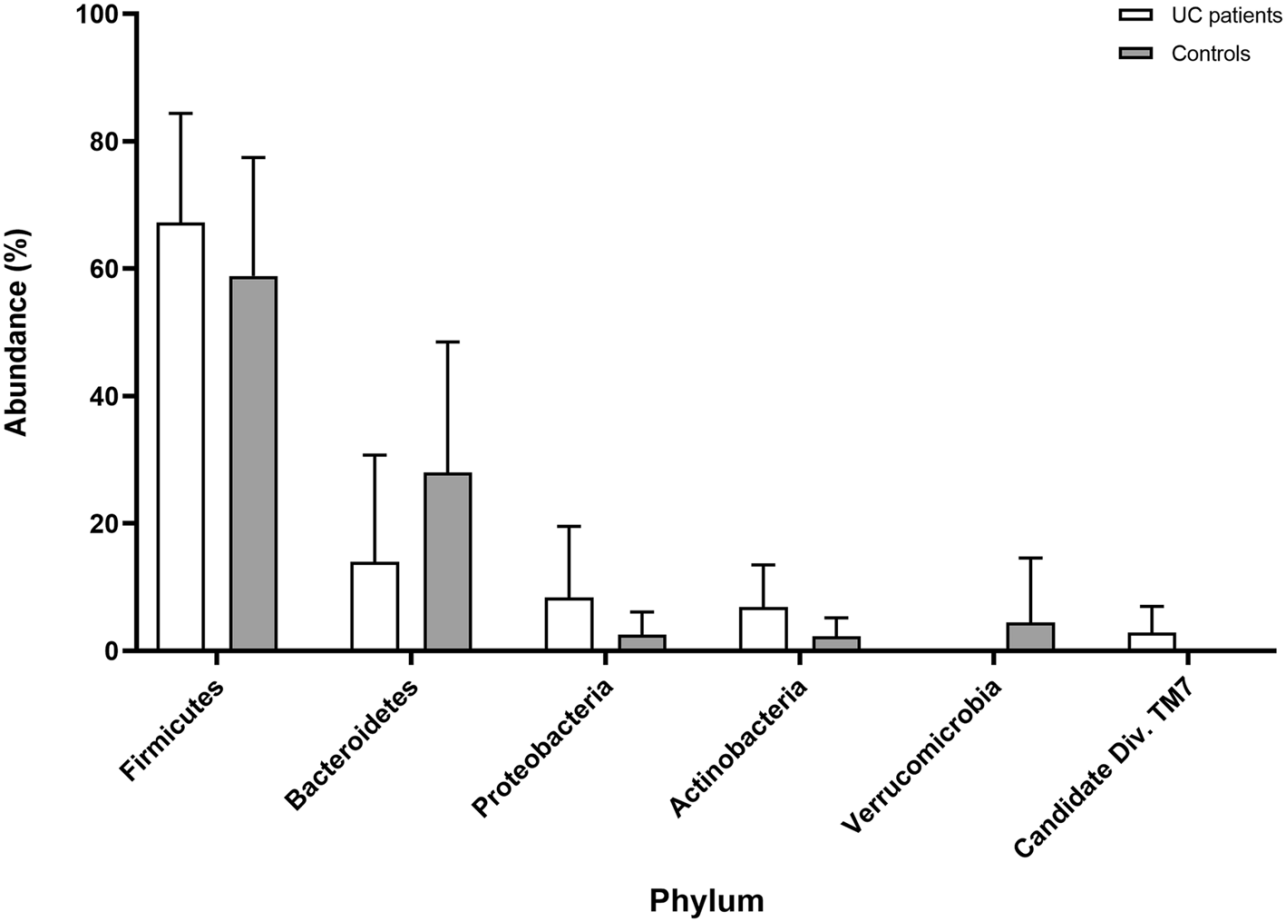
The main phylum level abundance in the gut microbiota in UC patients and healthy controls (mean values with SD).

**Figure 4:**
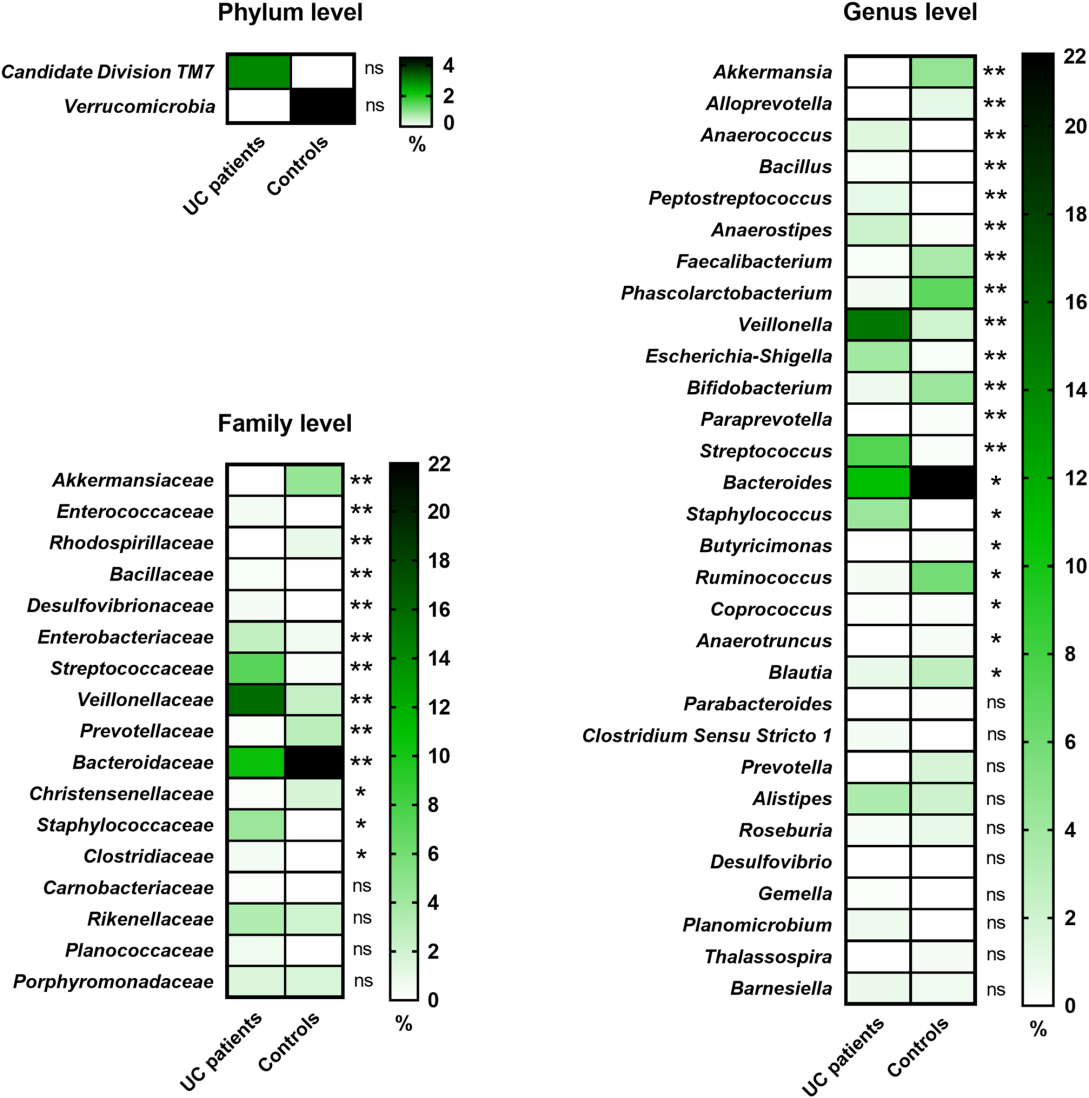
Differences in microbial composition between UC patients and healthy controls at phylum, family and genus level (the fill color correspond to the mean abundance value). These data only concern initial statistically significant results calculated using the non-parametric Mann–Whitney test and asterisks mark statistical significance obtained after multiple hypothesis testing correction according to the Benjamini–Hochberg procedure. ^**^p < 0.01, ^*^p < 0.05, ns—no significance.

The comparison of microbial abundance between UC patients and healthy controls was performed using Mann–Whitney test. Analysis at the phylum level showed that samples of UC patients are more abundant in *Proteobacteria* (8.42%) and *Actinobacteria* (6.89%) than those of healthy volunteers (2.57% and 2.29%, respectively) (Fig. 3). *Bacteroidetes* was presented at a lower level in the patient group (14%) relative to the controls (27.97%). In both groups, most of the bacteria identified belonged to *Firmicutes* phylum and constituted 67.24% in patients and 58.85% in control subjects. These four main phyla including *Firmicutes*, *Bacteroidetes*, *Actinobacteria* and *Proteobacteria* comprised over 90% of the total microbial community in analysed guts, although the observed differences were not statistically significant. Among the other things, less common phyla, *Verrucomicrobia*, *Candidate Division TM7*, *Euryarchaeota, Cyanobacteria* and *Synergistetes* were detected in the samples analysed. An abundance of *Verrucomicrobia* was observed to be significantly higher in healthy subjects relative to UC patients (4.47% and 0%, respectively, *p* = 0.04), but after multiple correction, the statistical significance was not high (Fig. 4) (detailed values shown in Supplementary Table S1). Furthermore, *Candidate Division TM7* was detected only in the patient group with a frequency of 2.88%, but any significant difference (*p* = 0.006) was also eradicated after correction (Fig. 4) (Supplementary Table S1).

At the family level, we observed that 13 bacterial taxa displayed significantly different abundance (*p* < 0.05) in both groups of samples (Fig. 4). All calculated values are presented in Supplementary Table S2. In UC patients, the dominant bacterial families were *Veillonellaceae* and *Ruminococcaceae,* making up 15.8% and 14.6%, respectively, whereas in healthy subjects *Ruminococcaceae* (29.7%) and *Bacteroidaceae* (21.9%) were found to be most abundant.

At the genus level, finally 20 bacterial taxa were at significantly different (*p* < 0.05) abundance level in patients with UC compared to healthy volunteers (Fig. 4) (Supplementary Table S3). Among considerable enriched genera in samples from UC patients were *Escherichia-Shigella*, *Peptostreptococcus*, *Bacillus*, *Veillonella*. The substantially decreased genera in those patients included *Akkermansia*, *Faecalibacterium* and *Bifidobacterium*.

### Differences in correlation between bacteria in analysed groups

**Figure 5:**
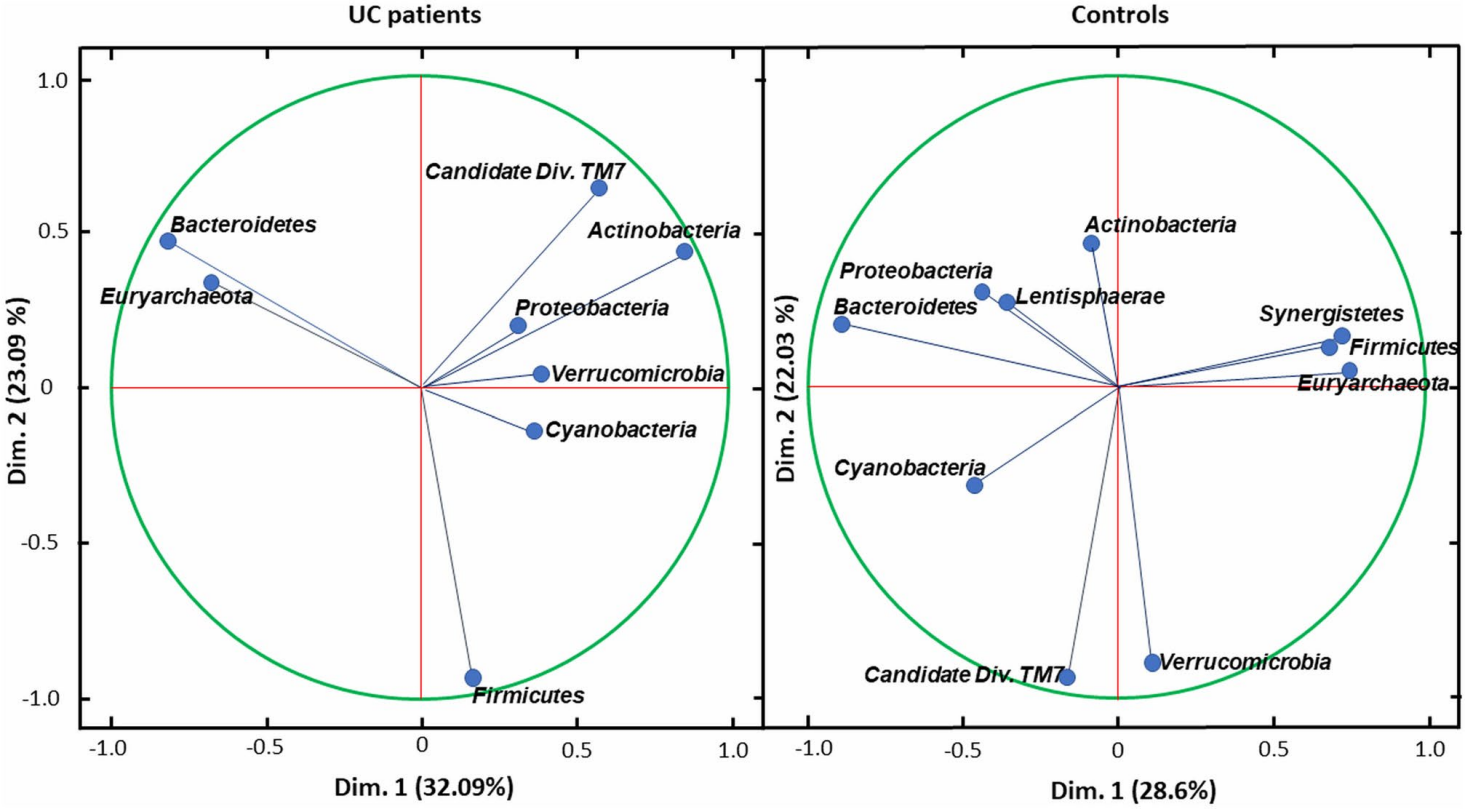
Projection of the variables at phylum level on the two dimensions biplot for UC patients (left) and controls (right).

In order to investigate the correlations between bacteria at the phylum level for UC patients and controls, principal component analysis (PCA) in two dimensions (dim.) was performed. This analysis demonstrated that in UC patient group *Bacteroidetes* and *Euryarchaeota* richness are strongly correlated with each other. A similar association was found for *Candidate Division TM7* and *Actinobacteria*. In contrast to these results, *Bacteroidetes* and *Euryarchaeota* in the control group showed the opposite correlation. Moreover, for the healthy subjects group the abundance of *Firmicutes*, *Synergistetes* and *Euryarchaeota,* as well as *Candidate Division TM7* with *Verrucomicrobia* are in strong correlation, which was not observed in UC patients (Fig. 5).

### Meta-analysis

**Figure 6:**
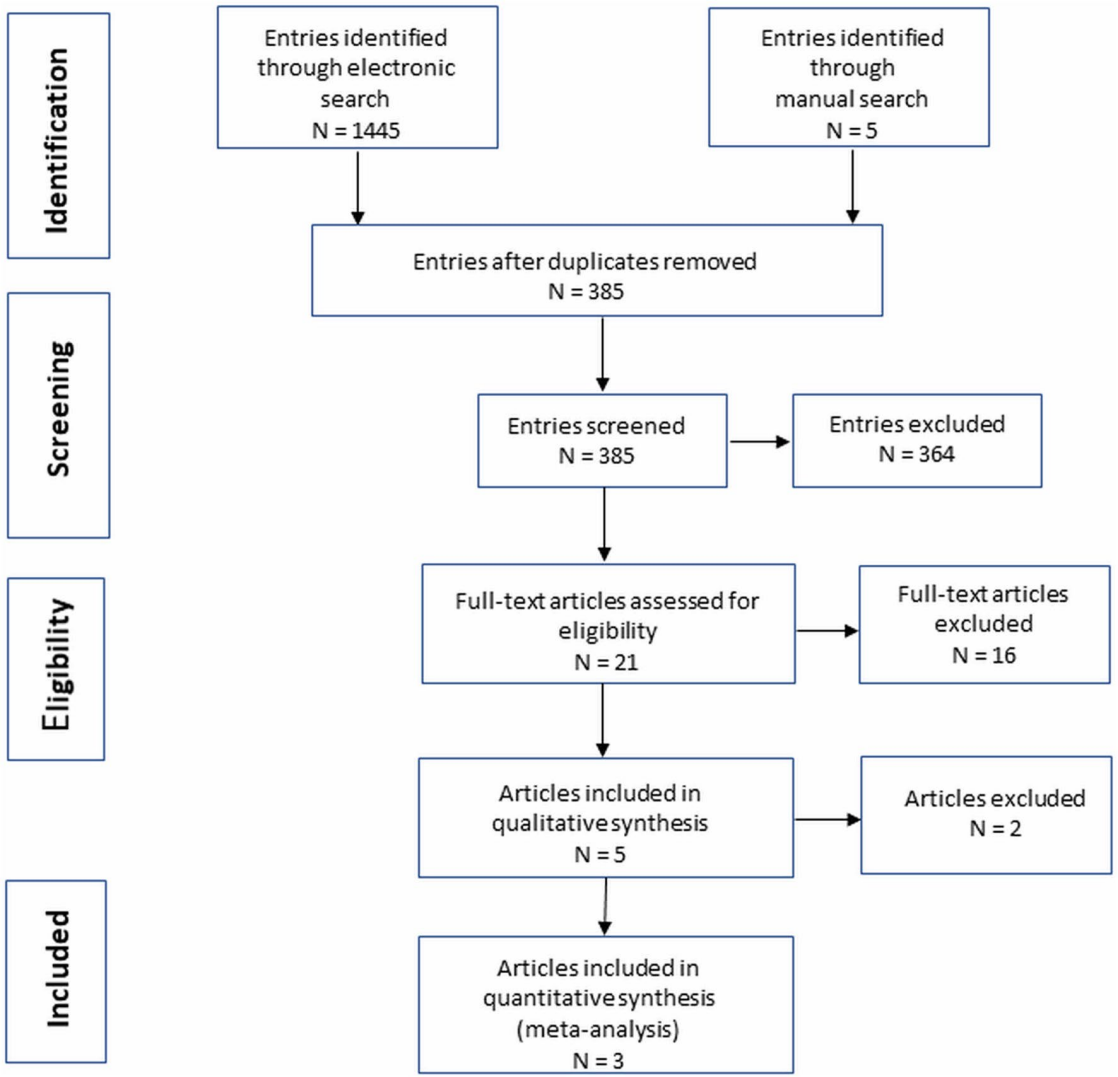
Flowchart of literature search.

Our electronic search identified 1445 potentially eligible entries (Fig. 6). Five additional records were found by the manual search in other sources. After filtering and excluding duplicates, a total of 21 items with full text availability were reviewed. Among these results, only 5 were compliant with our methodology (V3-V4 NGS) and the type of samples tested (faeces) in the comparative studies of UC patients with a control group^35–39^**.** Two of these studies did not report all quantitative data (mean values with standard deviation) about microbial abundance, as well as raw sequencing data were not available, even on request, which made it impossible to include them in the meta-analysis^38,39^.

Finally, we were able to include 3 studies in meta-analysis to verify our significant differences in microbiota composition between UC and control group^35–37^. The studies achieved relatively high scores in the quality assessment (NOS score) (seven to eight). Included studies are characterized in Table 2.

**Table 2:** Characteristics of included studies.

Study	UC patients (n)	Healthy controls (n)	Population	NOS^#^
Own research	10	10	Polish	7
Bajer 2017^35^	32	31	Czech	7
Alam 2020^36^	11	10	English	7
Clooney 2020^37^	228	161	Canadian and Irish	7

^#^Newcastle Ottawa scale (NOS) used to assess the quality of bias control.

Performed by us meta-analysis showed a significant difference in the content of several bacterial families and genera between the studied group of patients with UC and the controls.

### Streptococcaceae

**Figure 7:**
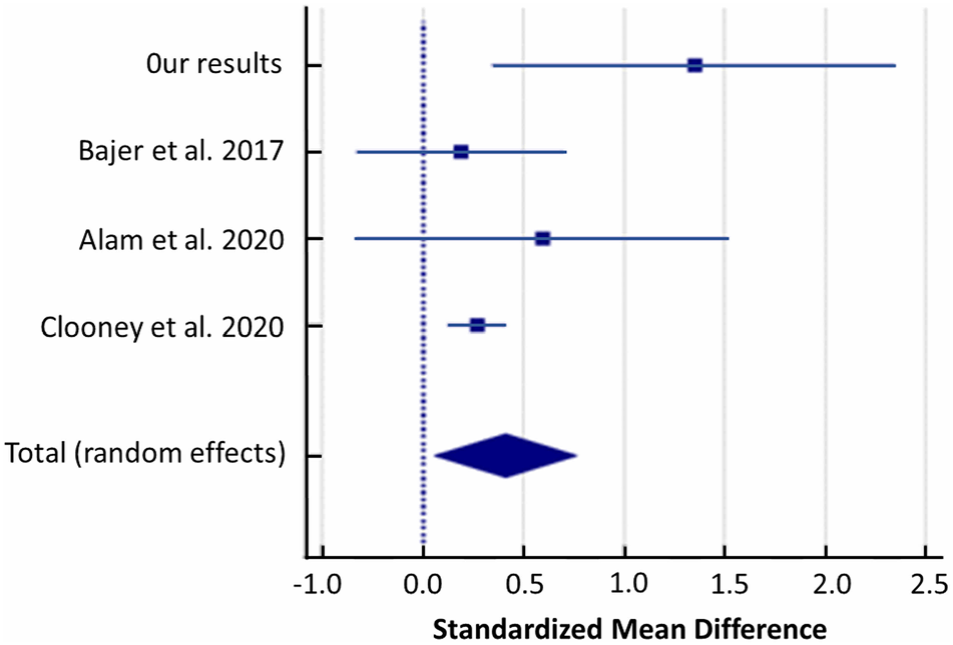
Forest plot of random-effects meta-analysis including studies evaluating the abundance of *Streptococcaceae* in UC patients versus healthy controls.

Random-effects meta-analysis of included studies evaluating gut microbiota showed that UC patients had a higher abundance of *Streptococcaceae* than healthy controls (SMD = 0.413, 95% CI: 0.0599 to 0.766; I^2^ = 46.40%; Fig. 7).

### Christensenellaceae

**Figure 8:**
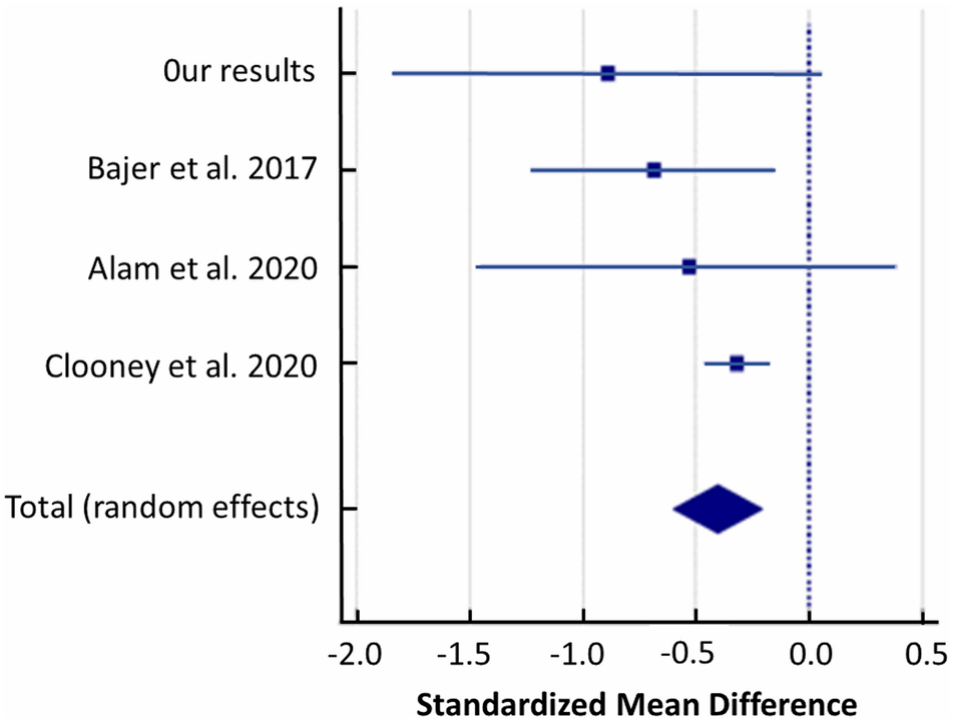
Forest plot of random-effects meta-analysis including studies evaluating the abundance of *Christensenellaceae* in UC patients versus healthy controls.

A random-effects meta-analysis of the studies showed that patients with UC had a lower abundance of *Christensenellaceae* compared to controls with a SMD of − 0.404 (95% CI − 0.602 to − 0.206) and heterogeneity (I^2^ = 10.07%) (Fig. 8).

### Phascolarctobacterium

**Figure 9:**
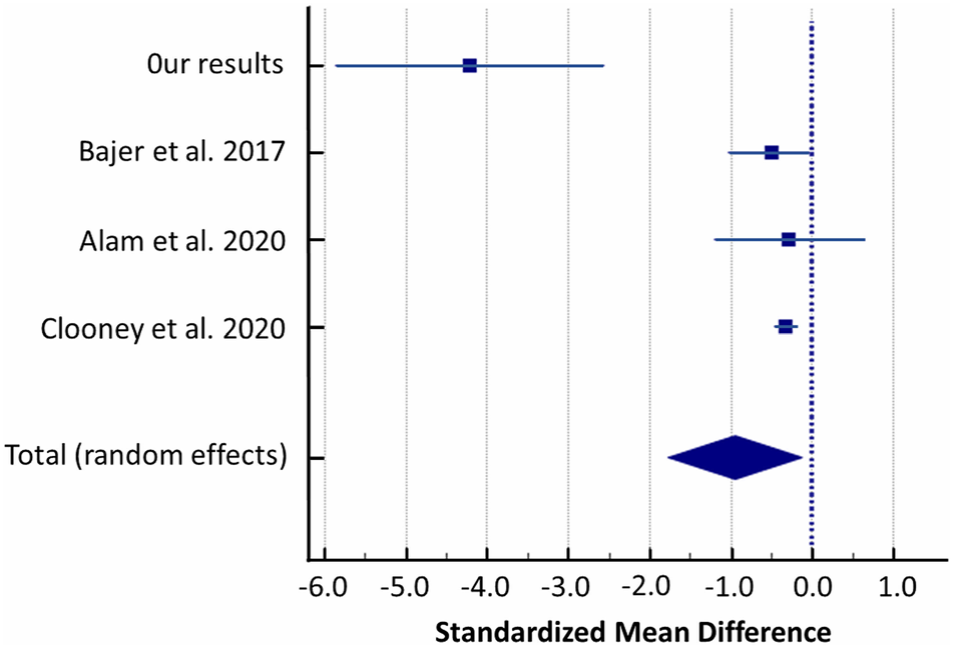
Forest plot of random-effects meta-analysis including studies evaluating the abundance of *Phascolarctobacterium* in UC patients versus healthy controls.

Random-effects meta-analysis revealed difference in the abundance of *Phascolarctobacterium* in patients with UC compared to healthy controls. *Phascolarctobacterium* was reduced in UC patients compared to controls (SMD = − 0.948, 95% CI − 1.772 to − 0.124; I^2^ = 87.63%; Fig. 9).

### Anaerostipes

**Figure 10:**
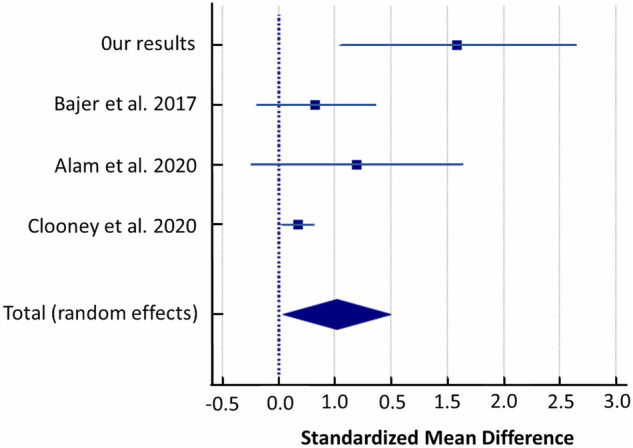
Forest plot of random-effects meta-analysis including studies evaluating the abundance of *Anaerostipes* in UC patients versus healthy controls.

Random-effects meta-analysis of the studies evaluating intestinal microbiota showed that UC patients had a higher abundance of this bacterial genus than healthy controls (SMD = 0.520, 95% CI: 0.0403 to 0.999; I^2^ = 67.81%; Fig. 10).

### Streptococcus

**Figure 11:**
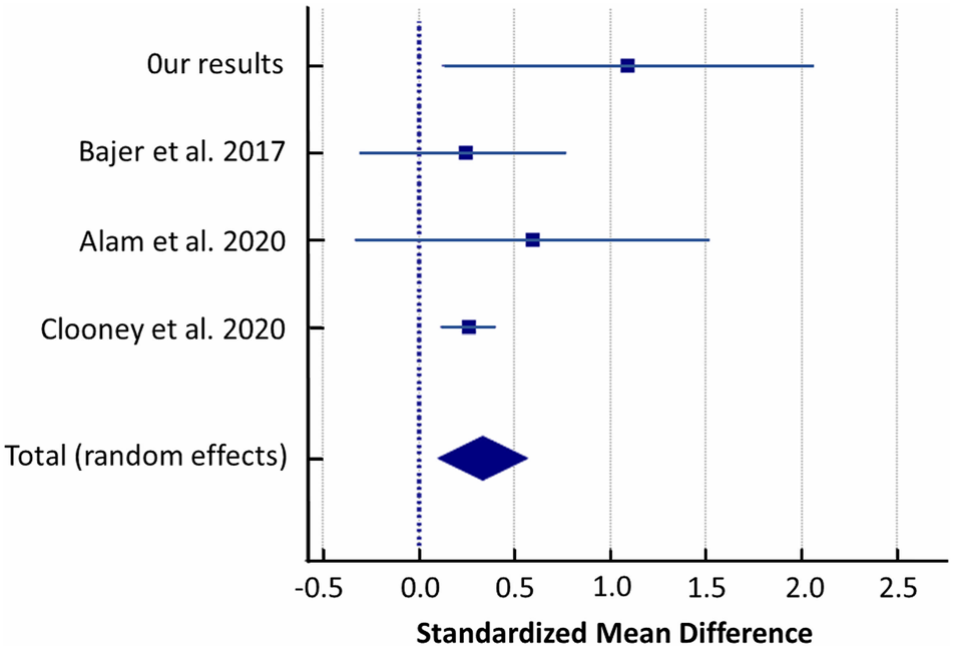
Forest plot of random-effects meta-analysis including studies evaluating the abundance of *Streptococcus* in UC patients versus healthy controls.

A random-effects meta-analysis of the studies showed that patients with UC had a higher abundance of *Streptococcus* compared to controls with a SMD of 0.336 (95% CI: 0.103 to 0.569) and heterogeneity (I^2^ = 17.94%) (Fig. 11).

### Ruminococcus

**Figure 12:**
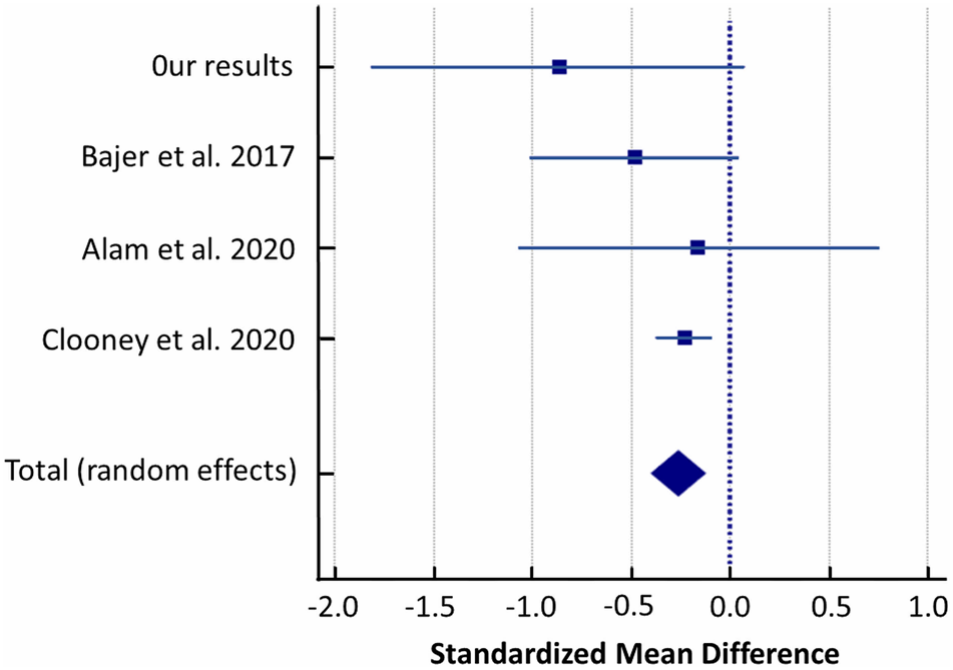
Forest plot of random-effects meta-analysis including studies evaluating the abundance of *Ruminococcus* in UC patients versus healthy controls.

A random-effects meta-analysis of included studies showed that patients with UC had a reduced abundance of *Ruminococcus* compared to controls with a SMD of − 0.261 (95% CI − 0.397 to -0.125) and heterogeneity (I^2^ = 0%) (Fig. 12).

## Discussion

The crucial role of intestinal microbiome in human health and disease has been discovered over last years. Studies highlight disturbances in the gut microbiological balance in IBD, although the results are not consistent^15^. The current study aimed to indicate the specific changes in the gut microbiota for Polish patients with UC compared to healthy volunteers. Our results demonstrated substantial quantitative and qualitative changes in the gut microbial composition in both groups of subjects.

We revealed that the diversity of microbiota in UC patients was reduced, which was manifested by the lower amount of total OTUs (*p* < 0.05), as well as Shannon’s entropy (even *p* > 0.05) compared to the control samples. We have identified on average 225 OTUs in healthy controls and 144 in UC subjects. These results confirm previous findings about dysbiosis observed in UC^12,40^.

This study shows that four basic bacterial phyla, including *Firmicutes*, *Bacteroidetes*, *Proteobacteria* and *Actinobacteria* comprised more than 90% of the intestinal microbiota in both groups of samples^41^. We have demonstrated, that *Firmicutes* were the most common phylum in both groups, with a mean frequency of 67.24% in patients and 58.85% in control subjects. This outcome seems to be rather surprising. An extensive literature review by Canadian researchers demonstrated substantially lower, or less often, a higher level of *Firmicutes* in patients with UC^28^. It is due to the important role of this bacterial phylum, particularly SCFAs production, of which butyrate is the main nutrition source for cells of the gut epithelium and its depletion, also reported in the case of UC, is associated with impairments in the gut barrier integrity^33^. On the other hand, a large inter-individual variability in the abundance of *Firmicutes* in healthy subjects, as well as in UC patients, has been observed in many studies of the intestinal microbiome, and also in our analysis (28–95%) (Fig. 2)^42^. The second dominant phylum, *Bacteroidetes*, was reported in our patients with UC at a lower level (14%) compared to the controls (27.97%). Most research performed so far in UC proves a decrease in *Bacteroidetes* in UC cases^28,43–46^. However, there are also studies leading to the opposite conclusions, e.g. Fuentes et al*.* observed significantly higher levels of *Bacteroidetes* in UC patients at baseline (before FMT) compared with donors, where differences ranged from 1.2 up to 15 times as much depending on the species^47^. We also have shown that samples from patients with UC are more abundant in *Proteobacteria* (8.42%) and *Actinobacteria* (6.89%) relative to healthy volunteers (2.57% and 2.29%, respectively), which correlates with the results of American scientists for 61 UC patients and 61 controls^43^. Furthermore, in our research, *Verrucomicrobia* was found to be more abundant in healthy subjects compared to UC. Despite the lack of statistically significant quantitative variations in the gut microbiota at the phylum level between both groups, considerable qualitative and quantitative differences in microbial compositions within phylas were identified.

We demonstrated that diversity in bacterial composition among *Firmicutes* concerned a considerable increase in *Bacillaceae*, *Clostridiaceae 1*, *Enterococcaceae*, *Staphylococcaceae*, *Streptococcaceae* and *Veillonellaceae,* as well as a decrease in the *Christensenellaceae* family in UC patients in relation to healthy controls (Fig. 4) (Supplementary Table S2). In our UC samples, we observed a reduction in beneficial bacteria, such as *Faecalibacterium* and *Blautia* producing butyric acid, as well as *Ruminococcus* (Fig. 4) (Supplementary Table S3)*,* which is consistent with previous findings summarized by Nagao-Kitamoto & Kamada^44^. What consequences could this have for UC patients? It is well established that a decrease in *Faecalibacterium* (particularly *F. prausnitzii*) is characteristic for IBD, including UC, because of its role. *F. prausnitzii* produces a 15 kDa anti-inflammatory protein that inhibits the NF-κB pathway in intestinal epithelial cells and was shown to prevent UC in a mouse model^48^. On the other hand, an increase of *Steptococcus*, *Staphylococcus* and *Veillonella* was proved in our UC patients group, which was also the case in some other studies^45^*.* Moreover, some research indicates a decrease in *Clostridium cluster XIVa* and an upward trend in the *Clostridium cluster IX* in UC^49^. In our UC patient group, an increase in *Clostridium* was observed, albeit not a statistically significant one. We revealed that a characteristic decrease in *Bacteroidetes* in the case of UC mainly concerns a reduction in *Bacteroidaceae* (genus *Bacteroides)* and *Prevotellaceae* (genus *Paraprevotella*, *Alloprevotella*). These results also confirm those obtained by Nemoto et al*.*^46^. However, in another investigation, *Bacteroidetes* significantly increased in UC^47^. The growth in *Proteobacteria* in the intestinal microbiota of UC patients observed in our study was caused generally by an increased level of *Enterobacteriaceae* (genus *Escherichia-Shigella*)*.* It is therefore worth highlighting the evidence that *Escherichia-Shigella* is correlated with inflammation and in healthy humans should not exceed 0.5% of the gut microbiota^32^. In our data, it constituted 3.9% in UC patients and 0.3% in the controls. Furthermore, we observed a substantial decrease in beneficial *Bifidobacterium* in Polish UC patients. A considerable decrease in *Akkermansia* (phylum *Verrucomicrobia*) was also found in our research as associated with UC, which is consistent with major findings. Previous studies demonstrated the anti-inflammatory effect of *Akkermansia* derived vesicles, which could reduce the substance triggering colitis^50^. The *Akkermansia* level could be decreased due to reduced mucins in patients with UC, which constitute an energy source for this bacteria^51^. These relationships indicate important genus that should be focused on in the search for microbiota markers of active UC, its course and the effect of FMT treatment.

The difference in gut microbiome between UC patients and healthy controls is also confirmed in our study by principal component analysis, which showed the existence of a strong correlation between different microbial phylas in both groups of samples. The difference in the correlation structures between the UC and healthy cohorts may result from the change in the microbiota environment in the gut due to the disease. This analysis shows, that certain correlations between individual units of the microbiota are not constant and may strongly depend on a number of factors. These factors may include for example the intestinal environment, availability of specific endogenic substances, which are affected by the disease^12^. Also international study confirmed, that stability of gut microbiota in patients with IBD is lower^37^.

There are some limitations to our study. The methodology used in our analysis for microbial community characterization was based on common V3-V4 fragments of the 16S rRNA gene with using the MiSeq, Illumina platform. However, there is evidence that the variety level in different microbial taxa may depend on the regions analyzed (V1-V9) in 16S rRNA gene^28^. The use of primers specific for some groups of bacteria may result in limited targeting and in the detection of other microbial groups, which could restrict comparisons in results between studies. Furthermore, the methodology applied in our study did not allow us to identify many bacteria at species level, but at most at genus level. In our research, we took care to analyse a homogeneous group of UC patients, but most studies include a combination of patients with active disease and in remission. Previous reports proved that the gut microbial profile of UC patients in remission is close to the microbiota of healthy individuals^22^. We enrolled patients only with active UC. For example, our results showed that the *Bifidobacterium* to *Enterobacteriaceae* ratio, which for healthy subjects is above 1, was 0.28 for UC patients and reached 7 for the controls.

Secondly, the limitation of our pilot study is small groups size. Thus, we performed a meta-analysis to verify our findings with other similar studies applying exactly the same methodology pipeline in the comparative analysis of UC cases versus controls, which are only few. After re-analysis of raw sequencing data from finally 3 studies and our results (281 UC cases and 212 controls in total) and random-effects meta-analysis we revealed significant differences between both studied groups in the abundance of *Streptococcaceae, Christensenellaceae, Ruminococcus, Streptococcus, Anaerostipes* and *Phascolarctobacterium*^35–37^*.*

In conclusion, our study conducted on Polish patients allowed us to point out alterations specific for UC in the gut microbial community compared to healthy controls. These results indicate that gut microbiota in UC is less diverse and considerable abundance differences are observed at the phylum, family and genus level compared to a healthy intestine. We reveal that dysbiosis in UC is generally based on a reduction in beneficial bacteria and an increase in undesirable groups of them. However, it should be taken into account that a number of factors may influence the specific structure of gut microbiota, including host genetic factors, like the expression of relevant inflammatory proteins. In the future, studies on bacterial species, their function and correlation with host genetic factors will be more desirable in discovering UC etiology.

## Materials and methods

### Patients and control group

Ten Polish patients (5 female and 5 men with an average age of 47.4 and an average BMI of 22 kg/m^2^) with active UC and under the care of the H. Swiecicki Clinical Hospital at Poznan University of Medical Sciences and the Department of Gastroenterology, Dietetics and Internal Diseases at Poznan University of Medical Sciences in Poland were enrolled in this study. All participants provided written informed consent. This study was approved by the local Ethics Committee of Poznan University of Medical Sciences (resolution no. 1106/17 approved on 9 November 2017). All experiments were performed in accordance with the principles of the 1964 Declaration of Helsinki with its later amendments. Patients were recruited from January 2018 to December 2019. UC diagnosis was made based on clinical, endoscopic and histological data. Detailed characteristics for all patients including age, BMI, treatment, range of disease, disease severity (based on Truelove-Witts score) and duration of disease are presented in Table 1. Exclusion criteria were additional diseases (as e.g., *Clostridium difficile* infection, diabetes, cancers), pregnancy and intake of antibiotics or probiotics during the last 3 months.

The control group consisted of 10 healthy subjects (6 female and 4 men with the average age of 45.2 years and average BMI of 23.8 kg/m^2^) from Polish population. Exclusion criteria were antibiotics or probiotics intake and alcohol abuse during the last 6 months. The first morning faecal sample was taken from each participant and stored until analysis at -80ºC.

### 16S rRNA gene sequencing

Bacterial DNA was isolated from the faecal samples collected from each individuals using a Genomic Mini AX Bacteria + Kit (A&A Biotechnology, Gdansk, Poland), according to the manufacturer's protocol. The sequences of region V3-V4 of the 16S rRNA bacterial gene were amplified using barcoded primers with Illumina adapters and KAPA HiFi HotStart ReadyMix PCR Kit (KAPA Biosystems, Cape Town, South Africa). Bacterial libraries were prepared according to 16S Metagenomic Sequencing Library Preparation protocol (Part # 15,044,223 Rev. B, Illumina, San Diego, CA, USA). The final concentration of library was 8 pM. Bacteriophage *Φ-X174* (PhiX) control library was added to the concentration of 20%. Sequencing was performed on an Illumina MiSeq platform using a MiSeq Reagent Kit v3 (600 cycles).

### Bioinformatic analysis

16S rRNA gene sequences were obtained and processed using a CLC Genomic Workbench 8.5 and CLC Microbial Genomics Module 1.2. (Qiagen Bioinformatics, Aarhus, Denmark). The average number of reads per sample was 217,819. Reads were demultiplexed and trimmed. Chimeric sequences were removed and operational taxonomic units (OTUs) were clustered against the SILVA v119 97% 16S rDNA gene database^52^. Alpha diversity was determined using OTU richness and Shannon’s entropy. Raw demultiplexed sequencing data with sample annotations were deposited in the Short Read Archive database (SRA) (http://www.ncbi.nlm.nih.gov/bioproject/PRJNA679275).

### Statistical analysis

The differences in demographic data between the groups were analysed by the *t-*test for independent measures. The assumption of whether the data follow the normal distribution was evaluated using the Shapiro–Wilk test. Levene’s test was used to check the equality of variances. In case the data did not confirm the assumptions, the non-parametric Mann–Whitney test was used. Furthermore, a correction with respect to multiple hypothesis testing was performed. The adjusted p-values were calculated according to the Benjamini–Hochberg procedure. In order to investigate the relationship between particular microbial phylas, principal component analysis (PCA) was performed.

The statistical analysis was performed using STATA software (Stata statistical software: Release 15. College Station, TX: StataCorp LLC). All tests were considered significant at *p* < 0.05. GraphPad Prism 8.0.1 (GraphPad software, San Diego, CA, USA) and CLC Genomics Workbench software (Qiagen Bioinformatics, Aarhus, Denmark) was applied to prepare graphs.

### Search and selection of studies for integrative research

We conducted a systematic search of studies in PubMed, Embase, The Cochrane Library and Web of Science database included the keywords (‘Inflammatory Bowel Disease’ [Mesh] OR ‘IBD’ [Mesh] OR ‘Colitis’ [Mesh]) AND (‘Microbiota’ OR ‘Microbiome’ OR ‘Dysbiosis’ OR ‘Bacteria’ OR Microflora’ OR ‘Microbial’) with the following filters: last 10 years, Clinical Trial, Controlled Clinical Trial, Randomized Controlled Trial, Humans, Adult. We also performed manual searches. The last search update was November 2020.

Including criteria were: case–control studies with adult patients diagnosed for active UC based on endoscopy and histology, where healthy individuals without history of disease were used as controls; assessments of microbiota composition based on stool samples using NGS technology covering V3 and V4 regions of 16S rRNA gene; studies with publicly available raw 16S data for each case and control samples (mostly downloaded from online repositories, as SRA). The raw sequencing data have been re-analysed by us according to the same pipeline as our data.

### Data synthesis and meta-analysis

The methodological quality of randomized studies was assessed using the Newcastle—Ottawa quality assessment scale case–control studies (Table 2). The highest quality studies are awarded up to nine stars. The assessment was made independently by two authors (O.Z.-B., M.M.). To compare the results of UC patients vs. control, a meta-analysis using a random model was performed. As a measure of the effect, the standardized mean difference (SMD) with 95% confidence interval was used. As a measure of heterogeneity I^2^, statistics based on Q-Cochran test was used. The Publication bias was assessed by Egger’s test. The calculations were performed by MedCalc statistical software version 19.6 (MedCalc software Ltd, Ostend, Belgium; https://www.medcalc.org; 2020).

## Supplementary Information

Supplementary Information
