# Supplementary material for: Dysbiosis of gut microbiota in Polish patients with ulcerative colitis: a pilot study
Source: Sci Rep. 2021 Jan 25;11:2166. doi: 10.1038/s41598-021-81628-3 (PMC7835370; doi:10.1038/s41598-021-81628-3)
Supplement: Supplementary file 1 — Supplementary Information [file 41598_2021_81628_MOESM1_ESM.docx]

**Dysbiosis of gut microbiota in Polish patients with ulcerative colitis: a pilot study.**

Oliwia Zakerska-Banaszak^1*^, Hanna Tomczak^2,3#^, Marcin Gabryel^4#^, Alina Baturo^4^, Lukasz Wolko^5^, Michal Michalak^6^, Natalia Malinska^2^, Dorota Mankowska-Wierzbicka^4^, Piotr Eder^4^, Agnieszka Dobrowolska^4^, Ryszard Slomski^1^, Marzena Skrzypczak-Zielinska^1^

1. Institute of Human Genetics, Polish Academy of Sciences, Poznan, Poland
2. Central Microbiology Laboratory, H. Swiecicki Clinical Hospital at the Poznan University of Medical Sciences, Poznan, Poland
3. Department of Dermatology and Venereology, Poznan University of Medical Sciences, Poznan, Poland
4. Department of Gastroenterology, Dietetics and Internal Diseases, Poznan University of Medical Sciences, Poznan, Poland
5. Department of Biochemistry and Biotechnology, University of Life Sciences, Poznan, Poland
6. Department of Computer Science and Statistics, Poznan University of Medical Sciences, Poznan, Poland

^#^ These authors contributed equally to this work

***Corresponding author:**

Oliwia Zakerska-Banaszak, PhD

Institute of Human Genetics, Polish Academy of Sciences,

Strzeszynska 32, 60-479 Poznan, Poland

E-mail: o.zakerska.banaszak@gmail.com

Tel. +48 513 384 141

Fax. +48 61 823 32 35

**Supplementary Table Legends**

**Supplementary Table S1** Differences in gut microbial compositions between UC patients and healthy controls at phylum level.

**Supplementary Table S2** Differences in gut microbial compositions between UC patients and healthy controls at family level.

**Supplementary Table S3** Differences in gut microbial compositions between UC patients and healthy controls at genus level.

**Supplementary Table S1.** Differences in gut microbial compositions between UC patients and healthy controls at phylum level.

| **Phylum** | **UC patients** | | | **Controls** | | ***p*-value** | **corrected**  ***p*-value*** |
| --- | --- | --- | --- | --- | --- | --- | --- |
|  | Mean [%] | | SD [%] | Mean [%] | SD [%] |  |  |
| *Candidate Division TM7* | **2.88** | **↑** | 4.11 | **0.012** | 0.020 | **0.006** | **0.061** |
| *Verrucomicrobia* | **0.0005** | **↓** | 0.0017 | **4.46** | 10.12 | **0.040** | **0.168** |

Presented data concern only initially statistically significant results calculated using non-parametric Mann-Whitney test and asterisk mark statistical significance obtained after multiple hypothesis testing correction method Benjamini-Hochberg.

**Supplementary Table S2.** Differences in gut microbial compositions between UC patients and healthy controls at family level.

| **Family** | **UC patients** | | | **Controls** | | ***p*-value** | **corrected**  ***p*-value*** |
| --- | --- | --- | --- | --- | --- | --- | --- |
|  | Mean [%] | | SD [%] | Mean [%] | SD [%] |  |  |
| [*Akkermansiaceae*](https://www.google.pl/search?bih=607&biw=1280&hl=pl&sxsrf=ALeKk00Dn2Eo8TjHpYJdeU2XYQMycbkXzw:1585103336156&q=Akkermansiaceae&stick=H4sIAAAAAAAAAONgVuLVT9c3NEwuNsnOy0jKWcTK75idnVqUm5hXnJmYnJqYCgD5PScIIwAAAA&sa=X&ved=2ahUKEwiGrKKKyrToAhUusKQKHW25CZkQmxMoATAVegQIERAD) | **0.0005** | **↓** | 0.001 | **4.47** | 10.086 | **0.0001** | **0.0016** |
| *Enterococcaceae* | **0.488** | **↑** | 0.91 | **0.002** | 0.004 | **0.0001** | **0.0016** |
| *Rhodospirillaceae* | **0** | **↓** | 0 | **0.945** | 1.417 | **0.00006** | **0.0016** |
| *Bacillaceae* | **0.339** | **↑** | 0.479 | **0.001** | 0.004 | **0.0002** | **0.0016** |
| *Desulfovibrionaceae* | **0.416** | **↑** | 0.375 | **0.013** | 0.006 | **0.0002** | **0.0016** |
| *Enterobacteriaceae* | **2.573** | **↑** | 1.415 | **0.604** | 0.420 | **0.0002** | **0.0016** |
| *Streptococcaceae* | **7.174** | **↑** | 6.935 | **0.231** | 0.646 | **0.0002** | **0.0016** |
| *Veillonellaceae* | **15.806** | **↑** | 6.538 | **2.486** | 1.118 | **0.0002** | **0.0016** |
| *Prevotellaceae* | **0.127** | **↓** | 0.376 | **2.850** | 3.668 | **0.0005** | **0.0028** |
| *Bacteroidaceae* | **10.640** | **↓** | 2.004 | **21.976** | 8.096 | **0.0007** | **0.004** |
| *Christensenellaceae* | **0.147** | **↓** | 0.313 | **1.743** | 2.411 | **0.0026** | **0.012** |
| *Staphylococcaceae* | **4.240** | **↑** | 7.99 | **0.002** | 0.006 | **0.0031** | **0.013** |
| *Clostridiaceae 1* | **0.513** | **↑** | 0.941 | **0.010** | 0.012 | **0.01** | **0.041** |
| *Carnobacteriaceae* | **0.113** | **↑** | 0.171 | **0** | 0 | **0.014** | **0.053** |
| *Rikenellaceae* | **3.400** | **↑** | 7.173 | **2.086** | 2.744 | **0.029** | **0.09** |
| *Planococcaceae* | **0.690** | **↑** | 2.168 | **0** | 0 | **0.035** | **0.102** |
| *Porphyromonadaceae* | **1.48** | **↑** | 3.1 | **1.56** | 1.9 | **0.023** | **0.08** |

Presented data concern only initially statistically significant results calculated using non-parametric Mann-Whitney test and asterisk mark statistical significance obtained after multiple hypothesis testing correction method Benjamini-Hochberg.

**Supplementary Table S3.** Differences in gut microbial compositions between UC patients and healthy controls at genus level.

| **Genus** | **UC patients** | | | **Controls** | | ***p*-value** | **corrected**  ***p*-value*** |
| --- | --- | --- | --- | --- | --- | --- | --- |
|  | Mean [%] | | SD [%] | Mean [%] | SD [%] |  |  |
| *Akkermansia* | **0.0005** | **↓** | 0.001 | **4.47** | 10.086 | **0.000087** | **0.0022** |
| *Alloprevotella* | **0** | **↓** | 0 | **1.076** | 1.102 | **0.000064** | **0.0022** |
| *Anaerococcus* | **1.351** | **↑** | 1.733 | **0** | 0 | **0.000064** | **0.0022** |
| *Bacillus* | **0.331** | **↑** | 0.333 | **0.00012** | 0.004 | **0.0001** | **0.0022** |
| *Peptostreptococcus* | **0.988** | **↑** | 0.863 | **0.00016** | 0.006 | **0.0001** | **0.0022** |
| *Anaerostipes* | **2.248** | **↑** | 1.798 | **0.140** | 0.130 | **0.0001** | **0.0022** |
| *Faecalibacterium* | **0.302** | **↓** | 0.574 | **3.623** | 1.200 | **0.0002** | **0.0025** |
| *Phascolarctobacterium* | **0.585** | **↓** | 0.302 | **6.814** | 1.982 | **0.0002** | **0.0022** |
| *Veillonella* | **14.870** | ↑ | 3.644 | **1.960** | 0.610 | **0.0002** | **0.0022** |
| *Escherichia-Shigella* | **3.922** | ↑ | 1.899 | **0.326** | 0.666 | **0.0003** | **0.0032** |
| *Bifidobacterium* | **0.713** | **↓** | 0.707 | **4.188** | 3.469 | **0.0005** | **0.0051** |
| *Paraprevotella* | **0** | **↓** | 0 | **0.226** | 0.318 | **0.0007** | **0.006** |
| *Streptococcus* | **7.297** | ↑ | 8.711 | **0.231** | 0.645 | **0.0012** | **0.0096** |
| *Bacteroides* | **10.928** | ↑ | 4.137 | **21.645** | 9.085 | **0.0028** | **0.019** |
| *Staphylococcus* | **4.240** | ↑ | 7.990 | **0.002** | 0.006 | **0.0032** | **0.019** |
| *Butyricimonas* | **0.020** | **↓** | 0.055 | **0.150** | 0.178 | **0.0034** | **0.019** |
| *Ruminococcus* | **0.433** | **↓** | 0.852 | **5.853** | 8.439 | **0.0034** | **0.019** |
| *Coprococcus* | **0.094** | **↓** | 0.289 | **0.249** | 0.374 | **0.0043** | **0.023** |
| *Anaerotruncus* | **0.009** | **↓** | 0.017 | **0.361** | 0.625 | **0.0049** | **0.025** |
| *Blautia* | **0.905** | **↓** | 1.012 | **2.707** | 1.481 | **0.0091** | **0.044** |
| *Parabacteroides* | **0.039** | **↓** | 0.088 | **0.108** | 0.075 | **0.011** | **0.051** |
| *Clostridium Sensu Stricto 1* | **0.509** | ↑ | 0.938 | **0.006** | 0.011 | **0.012** | **0.055** |
| *Prevotella* | **0.007** | **↓** | 0.017 | **1.716** | 3.265 | **0.016** | **0.068** |
| *Alistipes* | **3.397** | ↑ | 7.167 | **2.069** | 2.754 | **0.029** | **0.117** |
| *Roseburia* | **0.348** | **↓** | 0.545 | **0.957** | 0.441 | **0.030** | **0.117** |
| *Desulfovibrio* | **0** | **↓** | 0 | **0.017** | 0.026 | **0.035** | **0.117** |
| *Gemella* | **0.134** | ↑ | 0.252 | **0** | 0 | **0.035** | **0.117** |
| *Planomicrobium* | **0.690** | ↑ | 2.168 | **0** | 0 | **0.035** | **0.117** |
| *Thalassospira* | **0** | **↓** | 0 | **0.411** | 1.055 | **0.035** | **0.117** |
| *Barnesiella* | **0.769** | ↑ | 1.634 | **0.624** | 0.532 | **0.043** | **0.139** |

Presented data concern only initially statistically significant results calculated using non-parametric Mann-Whitney test and asterisk mark statistical significance obtained after multiple hypothesis testing correction method Benjamini-Hochberg.
